# Facial grimace during speech in cleft lip and palate: a proposal for classification

**DOI:** 10.1590/2317-1782/20212021069

**Published:** 2022-01-07

**Authors:** Rafaeli Higa Scarmagnani, Ana Paula Fukushiro, Renata Paciello Yamashita

**Affiliations:** 1 Programa de Pós-graduação em Ciências da Reabilitação, Hospital de Reabilitação de Anomalias Craniofaciais – HRAC, Universidade de São Paulo – USP - Bauru (SP), Brasil.; 2 Laboratório de Fisiologia, Hospital de Reabilitação de Anomalias Craniofaciais – HRAC, Universidade de São Paulo – USP - Bauru (SP), Brasil.; 3 Departamento de Fonoaudiologia, Faculdade de Odontologia de Bauru – FOB, Universidade de São Paulo – USP - Bauru (SP), Brasil.

**Keywords:** Cleft Palate, Velopharyngeal Insufficiency, Rhinomanometry, Facial Grimace, Speech Perception

## Abstract

**Purpose:**

To investigate the effectiveness of a proposal for classification of facial grimace (FG) and its correlation with objective evaluation of velopharyngeal closure (VPC).

**Methods:**

Twenty individuals with repaired cleft lip and palate underwent velopharyngeal area measurement by means of rhinomanometry and speech sample recording. The FG was rated in two steps, by three speech-language pathologists. First the evaluators rated the FG using their own criteria as: 1= absent FG; 2=mild; 3=moderate; 4=severe. Subsequently, they were submitted to a training session that established the following FG rating criteria: 1=absent FG; 2=movement only of the nose or upper third of the face; 3=strong movement of the nose or upper third of the face; 4=movement of the nose and upper third of the face. The evaluators rated the FG using the established criteria. Intra- and inter-rater agreement were calculated using weighted Kappa coefficient. Correlation between the two stage ratings with the VPC was calculated by Spearman’s correlation coefficient.

**Results:**

In the first stage inter-rater agreement ranged from fair to substantial; in the second stage, from substantial to almost perfect. Intra-rater agreement ranged from moderate to almost perfect in the first stage, and from moderate to substantial in the second stage. The correlation between FG and velopharyngeal area was positive and significant in both stages.

**Conclusion:**

The proposed FG judgement proved to be effective in determining the symptom and reliable in diagnosing the severity of velopharyngeal dysfunction. The significant correlation between perceptual and instrumental methods suggests that FG can be used in predicting VPC.

## INTRODUCTION

One of the characteristics often found in individuals with cleft lip and palate and velopharyngeal dysfunction (VPD) is facial grimace (FG), an unintentional behavior of contracting the nasal valve or, in some cases, eyebrows and forehead, in an attempt to prevent nasal air emission in order to achieve velopharyngeal closure^(1)^. This symptom mainly accompanies the production of oral pressure sounds and, as the other passive speech symptoms, should be identified and classified by means of auditory-perceptual assessment, considered the gold standard for the diagnosis of VPD and, therefore, essential in clinical diagnosis. Despite the undeniable importance of the auditory-perceptual assessment, the diagnosis of the velopharyngeal function and, consequently, the definition of the appropriate treatment for each case also demands instrumental assessments. Rhinomanometry, also known as the pressure-flow technique, is one of the instrumental methods recommended to complement the VPD diagnosis. This method has been the target of several studies which aim at correlating its findings to perceptual speech characteristics, in an attempt to predict the velopharyngeal function^(2,3)^. Facial grimace, for instance, is a characteristic frequently evaluated by clinicians and researchers in the assessment of velopharyngeal function, for being considered a good indicator of velopharyngeal behavior^(4)^ and thus, part of several auditory-perceptual assessment protocols used both in clinical practice and research. The task of identifying the VPD based on speech symptoms perceptively evaluated is very useful in clinical practice, since it allows professionals to make inferences on the velopharyngeal function when there is no access to instrumental assessment. However, as far as is known, no report of well-established and standardized criteria for classification specifically of facial grimace has been found in the literature. Ordinal numerical scales are used, in general, in protocols for the assessment of this symptom, although the results reliability may be questionable due to the subjectivity inherent to this procedure. Another approach reported in the literature, is the representation of facial movements by means of numerical scores, without considering, however, the severity of the symptom^(5)^. Therefore, this finding makes the development of a proposal based on standardized and well-defined criteria for the classification of facial grimace of great importance for clinical practice and research related to the topic.

The study aimed to investigate the effectiveness of a facial grimace rating proposal based on standardized criteria and its correlation with the quantitative objective instrumental evaluation of velopharyngeal closure in individuals with repaired cleft lip and palate.

## METHODS

### Subjects

This study was approved by the Institutional Review Board (approval number: 2.251.973) and all participants signed an Informed Consent Form. Twenty-subjects with repaired cleft palate associated or not with cleft lip were evaluated, aged between 6 and 38 years.

Since the purpose of this study was to evaluate the effectiveness of a classification of facial grimace and its correlation with rhinomanometry, factors such as cleft type, type of surgical technique used in primary palatoplasty and patient age did not influence the results, therefore, there was no need for distribution of individuals into subgroups. Care was taken to include adults and children in the study in order to establish a comprehensive sample with regard to age range. The age of 6 years was adopted as the minimum age due to the fact that, in general, children from this age range on are capable of understanding the rhinomanometry procedure and collaborate to its performance.

Individuals with physical and/or mental incapacity to perform the tests, acute or chronic allergic respiratory symptoms resulting in nasal congestion during the test, nasal area values below the expected values for age verified in rhinomanometry, residual palate fistulas, presence of pharyngeal flap, and compensatory articulation in the production of the “p” consonant were not included.

### Procedures

I - Velopharyngeal orifice area measurement by means of rhinomanometry (pressure-flow technique): the velopharyngeal cross-sectional area was determined during the production of the “p” consonant inserted in the word “rampa”^(6)^. Based on the obtained orifice area values, the velopharyngeal closure (VC) was classified according to the following criteria adapted from the literature^(2)^: 0 to 4.9mm^2^=adequate; 5 to 19.9mm^2^=borderline; and > 20mm^2^=inadequate. The PERCI-SARS (Microtronics Corp, version 4.01) computerized system was used for that purpose.
II - Digital audiovisual recording of the speech sample composed of sentence reading or repetition: For the speech analysis of this study, 12 sentences composed of 12 pressure consonants (target consonants) spoken in Brazilian Portuguese were used^(7)^. Both procedures (audiovisual recording and rhinomanometry) were performed on the same day. For the video recording, JVC digital camcorder (model GZ-MG555) was used supported by a tripod positioned one meter away from the patient.
III - Analysis of speech samples for rating facial grimace: the FG rating was carried out by 3 speech-language pathologists with an average of 7 years experience in treating individuals with cleft lip and palate. The perceptual judgment occurred in two stages. In the first one, the evaluators rated the facial grimace in 20 speech samples. The three raters were instructed to rate the FG based on their own criteria, the same criteria routinely used in clinical practice, using a 4-point ordinal scale with which all raters were familiar: 1=absent facial grimace; 2=mild grimace; 3=moderate grimace; 4=severe grimace. One week later, the raters were submitted to a training in which, through pictures and videos, the new criteria to be used for rating the facial grimace in the second stage were introduced and exemplified, namely: 1=absence of facial grimace; 2=movement only of the nose or upper third of the face; 3=strong movement of the nose or upper third of the face; 4=movement of the nose and upper third of the face.

After training, the raters carried out the second stage of evaluation, classifying FG on the same samples using the newly established criteria. The samples were distributed to the raters in portable memory devices (USB flash drives) and half of them were resubmitted two days on average after the end of each stage for intra-rater analysis. Thus, each rater analyzed a total of 60 speech samples. In both stages, the videos were edited and presented to the raters without the audio resource, to eliminate any type of interference and/or influence of speech resonance and other auditory symptoms of VPD during facial grimace analysis.

### Statistical analysis

Inter- and intra-rater agreement was established using the weighted Kappa coefficient following criteria determined for the interpretation of indexes^(8)^ and the comparison between the two stages was performed employing the chi-square test. To investigate the correlation between velopharyngeal orifice size and facial grimace rating, the mode of the scores was calculated given by the raters for facial grimace in the first and second stages. The correlation between the mode of the perceptual judgment of the facial grimace in both stages and the velopharyngeal orifice size determined by the instrumental evaluation was analyzed utilizing Spearman's Correlation Coefficient following criteria for its interpretation^(9)^ and considering a 5% significance level.

## RESULTS

### Inter- and intra-rater agreement

In the first stage, in which the raters rated facial grimace using their own criteria, inter-rater agreement ranged from fair (0.24) to substantial (0.62). In the second stage, in which the raters rated the grimace according to the criteria defined during training, agreement ranged from substantial (0.66) to almost perfect (0.80), as shown in Table 1. As for the intra-rater agreement, in the first stage, it varied from moderate (0.49) to almost perfect (1.0) while in the second stage this index varied from moderate (0.52) to substantial (0.67), as shown in Table 2.

**Table 1:** Inter-rater agreement in the first and second stages: kappa index, percentage of agreement and interpretation of the result

Raters		First Stage		Second Stage		
	Kappa Index	%	Interpretation	Kappa Index	%	Interpretation
1 and 2	0.62	75	Substantial	0.66	75	Substantial
1 and 3	0.24	50	Fair	0.80	85	Almost Perfect
2 and 3	0.33	55	Fair	0.73	80	Substantial

**Table 2:** Intra-rater agreement in the first and second stages: kappa index, percentage of agreement and interpretation of the result

Raters		First Stage		Second Stage		
	Kappa Index	%	Interpretation	Kappa Index	%	Interpretation
1	1.0	100	Almost Perfect	0.58	70	Moderate
2	1.0	100	Almost Perfect	0.67	80	Substantial
3	0.49	70	Moderate	0.52	70	Moderate

### Comparative analysis of the inter- and intra-rater agreement between the first and second stages

The inter-rater agreement concerning FG classification obtained in the second stage was higher than that observed in the first stage, although without significance.

The comparison between both judgments by the same rater, there was an increase in the kappa index for rater 3, but a decrease in this index for raters 1 and 2. The differences were not significant.

### Correlation between velopharyngeal orifice size and facial grimace

Spearman's correlation coefficient analysis revealed a moderate significant positive correlation between facial grimace and the velopharyngeal orifice area measurement in both the first stage of the analysis (*p*<0.01; r=0.550) and the second stage (*p*<0.01; r=0.553).

## DISCUSSION

In clinical practice, as well as in well-designed research, countless trials have been increasingly tested in an attempt to develop methods that result in higher agreement and reliability of results, especially in procedures involving perceptual assessments of a subjective nature. Special attention is given to the methods of classification and types of assessment scales of speech symptoms such as resonance, intelligibility, vocal quality, and articulation^(2,10-14)^.

Despite of great importance in the speech assessment of individuals with VPD, the same emphasis is not extended to facial grimace. Although it is an important indicator of VPD, no proposal for classification of facial grimace has so far been published in the literature. Its analysis is based on direct visualization of the speaker and is usually classified by equal interval scales or binary scales, consisting of presence or absence^(15)^.

The facial grimace rating proposal used in the present study was based on the clinical experience of its authors. A brief explanation was carried out considering the criteria used for the analysis of the samples in the second stage of the study to verify their influence on the results.

When analyzing the results of the intra-rater agreement, after the presentation of this new proposal, a reduction in the agreement index was observed. Several internal and external factors may interfere with subjective judgments such as the one in the present study. This is why perceptual assessment is subject to variability even when it is carried out by experienced raters. The physical and emotional state when assessing the samples, as well as the internal standard of each evaluator, which is also unstable, may have contributed to the variability of responses, even in the case of evaluators experienced in the evaluation and treatment of patients with CLP. It is known that the evaluator's internal standard is developed along his/her experience with the severity of the speech symptoms and this information is stored in memory^(10)^. It is speculated that, when facing a proposal that is different from the one usually used and more careful as to the location of the compensatory movement, the internal standards acquired along with their experience (concept of mild, moderate and severe symptom) for this type of assessment, ended up aggregating to the new way of evaluating, making it difficult to repeat the answers.

The same, however, did not occur with the inter-rater analysis. In this analysis, it was verified that the agreement index increased considerably after the presentation of the classification proposal, which speaks in favor of the new approach, especially for use in clinical studies and inter-center result assessment.

This higher agreement after training can be explained by the fact that in this analysis the evaluators had to use the pre-established reference descriptions, that is, they had a model to be followed during the assessments. This, added to the fact that the raters performed the rating right after training, attests to the use of these procedures in subjective analyses.

The fact that the raters were not trained and no criteria for the analysis of facial grimace were pre-established in the first stage of the study may be considered a limitation of the study. This methodology was adopted in order to avoid the influence of prior training on the results of the evaluation performed according to the internal standards and criteria used in the clinical routine of the evaluators.

The correlation of the facial grimace judgment with the velopharyngeal orifice dimension measured by the objective instrumental evaluation, in both stages, proved to be positive and significant, indicating that the higher the movement intensity during facial grimace, the higher the velopharyngeal gap size.

The use of rhinomanometry for evaluating VPD, as it is an objective method that determines the real size of the velopharyngeal gap is of great importance in clinical practice. For that reason, a group of researchers in Brazil has used this instrument to elaborate proposals of speech characteristic assessment protocols of patients with VPD, whose results are based on information also coming from this instrument^(2,3)^. It is expected, with the results of these studies, that professionals who do not have access to this kind of equipment may, based on the perceptual speech characteristics, be able to predict the size of the velopharyngeal gap, thus allowing safer and more effective conduct.

## CONCLUSION

The proposal of visual facial grimace judgment based on standardized criteria proved to be effective in determining the symptom and reliable in diagnosing the severity of VPD. The significant correlation between perceptual and instrumental evaluation methods suggests that facial grimace can be used to predict velopharyngeal closure.
