# Supplementary material for: Facial grimace during speech in cleft lip and palate: a proposal for classification
Source: Codas. 2022 Jan 7;34(3):e20210069. doi: 10.1590/2317-1782/20212021069 (PMC9769419; doi:10.1590/2317-1782/20212021069)
Supplement: Translation

# Translation: Mímica facial durante a fala em fissura labiopalatina: uma proposta de classificação

**Authors:** Rafaeli Higa Scarmagnani, Ana Paula Fukushiro, Renata Paciello Yamashita

**Affiliations:** 1 Programa de Pós-graduação em Ciências da Reabilitação, Hospital de Reabilitação de Anomalias Craniofaciais – HRAC, Universidade de São Paulo – USP - Bauru (SP), Brasil.; 2 Laboratório de Fisiologia, Hospital de Reabilitação de Anomalias Craniofaciais – HRAC, Universidade de São Paulo – USP - Bauru (SP), Brasil.; 3 Departamento de Fonoaudiologia, Faculdade de Odontologia de Bauru – FOB, Universidade de São Paulo – USP - Bauru (SP), Brasil.

**Keywords:** Fissura Palatina, Insuficiência Velofaríngea, Rinomanometria, Mímica Facial, Percepção da Fala

## Abstract

**Objetivo:**

Investigar a efetividade de proposta de classificação da mímica facial (MF), e sua correlação com avaliação objetiva do fechamento velofaríngeo (FVF).

**Método:**

Vinte indivíduos com fissura labiopalatina reparada foram submetidos à medida da área velofaríngea por meio da rinomanometria e à gravação de amostra de fala. A MF foi classificada em dois momentos, por três fonoaudiólogas. Inicialmente as avaliadoras classificaram a MF, utilizando critérios próprios, em: 1=MF ausente; 2=leve; 3=moderada; 4=grave. Posteriormente, foram submetidas a um treinamento com o estabelecimento dos seguintes critérios de classificação: 1=MF normal; 2=movimento somente de nariz ou terço superior da face; 3=movimento acentuado de nariz ou terço superior da face; 4=movimento de nariz e terço superior da face e, classificaram a MF utilizando os critérios estabelecidos. Concordância intra e interavaliadores foram calculadas pelo coeficiente Kappa ponderado. A correlação entre os resultados das etapas com o FVF foi feita pelo coeficiente de correlação de Spearman.

**Resultados:**

Na primeira etapa, a concordância interavaliadores variou de regular a substancial e na segunda, de substancial a quase perfeita. A concordância intra-avaliadores variou de moderada a quase perfeita na primeira etapa, e de moderada a substancial, na segunda etapa. A correlação entre a MF e área velofaríngea na primeira e na segunda etapa foi positiva e significativa.

**Conclusão:**

A proposta de julgamento da MF mostrou-se efetiva na determinação do sintoma e confiável no diagnóstico da gravidade da disfunção velofaríngea. A correlação significativa entre os métodos perceptivo e instrumental sugere que a MF pode ser utilizada na previsão do FVF.

## INTRODUÇÃO

Uma das características frequentemente encontradas em indivíduos com fissura labiopalatina e disfunção velofaríngea (DVF) é a mímica facial (MF), um comportamento não intencional de contrair a válvula nasal ou, em alguns casos, sobrancelhas e testa, na tentativa de impedir o escape de ar nasal, a fim de alcançar o fechamento velofaríngeo^(1)^. Esse tipo de sintoma acompanha, principalmente, a produção de sons orais de pressão da fala e deve ser, assim como os demais sintomas passivos de fala, identificado e classificado por meio da avaliação perceptivo-auditiva da fala, considerada padrão ouro para o diagnóstico da DVF e, portanto, essencial no diagnóstico clínico. Apesar da inegável importância da avaliação perceptivo-auditiva, o diagnóstico da função velofaríngea e, consequentemente, a definição de conduta terapêutica apropriada para cada caso exige, também, a avaliação instrumental. Dentre os métodos instrumentais recomendados para o complemento do diagnóstico da DVF está a rinomanometria, também conhecida como técnica fluxo-pressão. Este método tem sido alvo de diversos estudos que visam correlacionar os seus achados às características perceptivas da fala, na tentativa de, a partir destas características, prever a função velofaríngea^(2,3)^. A mímica facial, por exemplo, é uma característica frequentemente avaliada por clínicos e pesquisadores na avaliação da função velofaríngea, por ser considerada um bom indicador do comportamento velofaríngeo^(4)^ integrando, assim, diversos protocolos de avaliação perceptivo-auditiva da fala empregados tanto na prática clínica quanto em pesquisas. A tarefa de identificar a DVF com base nos sintomas de fala avaliados perceptivamente é bastante útil na prática clínica, pois, possibilita ao profissional fazer inferências sobre a função velofaríngea quando não se tem acesso a equipamentos para o diagnóstico instrumental. No entanto, até onde se sabe, nenhuma proposta com critérios bem estabelecidos e padronizados de classificação especificamente da mímica facial foi encontrada na literatura. O que se usa nos protocolos para a avaliação deste sintoma, de modo geral, são as escalas numéricas ordinais, para as quais, a confiabilidade dos resultados pode ser questionável devido à subjetividade inerente a este procedimento. Ou, ainda, como já proposto na literatura, a representação do movimento facial por meio de escores numéricos, sem considerar, entretanto, a gravidade do sintoma^(5)^. Tal constatação torna a elaboração de uma proposta baseada em critérios padronizados e bem definidos para a classificação da mímica facial, de grande importância para a prática clínica e pesquisas relacionadas ao tema.

Desse modo, este estudo teve como objetivo investigar a efetividade de uma proposta de classificação da mímica facial baseada em critérios padronizados e sua correlação com a avaliação instrumental objetiva quantitativa do fechamento velofaríngeo em indivíduos com fissura labiopalatina reparada.

## MÉTODO

### Casuística

Este estudo teve aprovação do Comitê de Ética em Pesquisa (número do parecer: 2.251.973) e todos os participantes assinaram o Termo de Consentimento Livre e Esclarecido. Foram avaliados 20 indivíduos com fissura palatina reparada associada ou não à fissura de lábio, com idade entre 6 e 38 anos.

Por tratar-se de um estudo cujo objetivo foi avaliar a efetividade de uma proposta de classificação da mímica facial e sua correlação com a rinomanometria, fatores como tipo de fissura, tipo de técnica cirúrgica utilizada na correção primária do palato e, idade do paciente não influenciaram os resultados, não havendo, portanto, necessidade de distribuição dos indivíduos em grupos distintos. Tomou-se o cuidado, inclusive, de incluir no estudo adultos e crianças a fim de se estabelecer uma amostra abrangente no que se refere à faixa etária. A idade de 6 anos foi adotada como idade mínima neste estudo pelo fato de que, crianças a partir desta faixa etária, de modo geral, são capazes de compreender o exame de rinomanometria e colaborar para a sua realização.

Não foram incluídos os indivíduos com incapacidade física e/ou mental para realizar os exames, sintomas respiratórios alérgicos agudos ou crônicos que resultassem em congestão nasal ao exame, valores de área nasal abaixo do esperado para a idade verificados na rinomanometria, fístulas residuais no palato, presença de retalho faríngeo e articulação compensatória na produção da consoante “p”.

### Procedimentos

I – Medida da área do orifício velofaríngeo por meio da rinomanometria (técnica fluxo-pressão): A área de secção transversa da velofaringe foi determinada durante a produção da consoante “p” inserida no vocábulo *“rampa”* ^(6)^. A partir dos valores de área obtidos, o fechamento velofaríngeo (FVF) foi classificado com base no seguinte critério adaptado da literatura^(2)^: 0 a 4,9mm^2^=fechamento velofaríngeo adequado; 5 a 19,9mm^2^=fechamento marginal e, >20mm^2^=fechamento inadequado. Para tanto foi utilizado o sistema computadorizado PERCI-SARS, versão 4.01 (Microtronics Corp.).
II - Gravação digital audiovisual da amostra de fala composta por leitura ou repetição de sentenças: Para a análise de fala deste estudo foram utilizadas 12 sentenças compostas por 12 consoantes de pressão (consoantes alvo) faladas no Português Brasileiro^(7)^. Ambos os procedimentos (gravação e rinomanometria) foram realizados no mesmo dia. Para o registro em vídeo, utilizou-se uma filmadora digital marca JVC, modelo GZ-MG555, sustentada por um tripé posicionado a um metro de distância do paciente.
III - Análise das amostras de fala para a classificação da mímica facial: A classificação da MF foi realizada por 3 fonoaudiólogas com experiência de 7 anos, em média, no tratamento de indivíduos com fissura labiopalatina. O julgamento perceptivo ocorreu em duas etapas. Na primeira etapa, as avaliadoras classificaram a mímica facial em 20 amostras de fala. Para tal, as três avaliadoras foram orientadas a classificar a MF com base em seus próprios critérios, os mesmos utilizados de rotina na prática clínica e utilizando, para tanto, a escala ordinal de 4 pontos, que todas as avaliadoras estavam familiarizadas: 1=mímica facial ausente; 2=mímica leve; 3=mímica moderada; 4=mímica grave. Uma semana depois, as avaliadoras foram submetidas a um treinamento no qual, por meio de figuras e vídeos, foram introduzidos e exemplificados os novos critérios a serem utilizados para a classificação da mímica facial na segunda etapa de avaliação, quais sejam: 1=ausência de mímica facial; 2=movimento somente de nariz ou terço superior da face; 3=movimento acentuado de nariz ou terço superior da face; 4=movimento de nariz e terço superior da face.

Após o treinamento, as avaliadoras realizaram a segunda etapa de avaliação classificando, nas mesmas amostras, a mímica facial utilizando os novos critérios estabelecidos. As amostras foram distribuídas às avaliadoras em dispositivos de memória portátil (*pen-drives*) e metade delas foi reapresentada dois dias, em média, após o final de cada etapa para a análise intra-avaliadores. Assim, cada avaliadora analisou um total de 60 amostras de fala. Em ambas as etapas, os vídeos foram editados e apresentados às avaliadoras sem o recurso do áudio, a fim de eliminar qualquer tipo de interferência e/ou influência da ressonância de fala e outros sintomas auditivos da DVF durante a análise da mímica facial.

### Análise estatística

As concordâncias inter e intra-avaliadores foram estabelecidas por meio do coeficiente Kappa ponderado seguindo critérios determinados para a interpretação dos índices^(8)^ e a comparação entre as duas etapas foi realizada por meio do teste qui-quadrado. Para investigar a correlação entre o tamanho do orifício velofaríngeo e a classificação da mímica facial, calculou-se a moda dos escores atribuídos pelas avaliadoras para a mímica facial na primeira e segunda etapas. A correlação entre a moda do julgamento perceptivo da mímica facial de ambas as etapas e o tamanho do orifício velofaríngeo determinado pela avaliação instrumental foi analisada por meio do Coeficiente de Correlação de Spearman seguindo critérios para sua interpretação^(9)^ e considerando-se o nível de significância de 5%.

## RESULTADOS

### Concordância Inter e Intra-avaliadores

Na primeira etapa, na qual as avaliadoras classificaram a mímica facial utilizando seus próprios critérios, a concordância interavaliadores variou de regular (0,24) a substancial (0,62). Na segunda etapa, na qual as avaliadoras classificaram a mímica de acordo com os critérios definidos durante o treinamento, a concordância variou de substancial (0,66) a quase perfeita (0,80), conforme apresentado na Tabela 1. Quanto à concordância intra-avaliadores, na primeira etapa, variou de moderada a (0,49) a quase perfeita (1,0) enquanto que, na segunda etapa esse índice variou de moderada (0,52) a substancial (0,67), como mostra a Tabela 2.

**Tabela 1:** Concordância interavaliadores na primeira e segunda etapa: índice kappa, porcentagem de concordância e interpretação do resultado

Avaliadoras		Primeira Etapa		Segunda Etapa		
	Índice Kappa	%	Interpretação	Índice Kappa	%	Interpretação
1 e 2	0,62	75	Substancial	0,66	75	Substancial
1 e 3	0,24	50	Regular	0,80	85	Quase Perfeita
2 e 3	0,33	55	Regular	0,73	80	Substancial

**Tabela 2:** Concordância intra-avaliadores na primeira e segunda etapa: índice kappa, porcentagem de concordância e interpretação do resultado

Avaliadoras		Primeira Etapa		Segunda Etapa		
	Índice Kappa	%	Interpretação	Índice Kappa	%	Interpretação
1	1,0	100	Quase perfeita	0,58	70	Moderada
2	1,0	100	Quase Perfeita	0,67	80	Substancial
3	0,49	70	Moderada	0,52	70	Moderada

### Análise comparativa da concordância inter e intra-avaliadores entre a primeira e a segunda etapa

O índice de concordância entre as avaliadoras quanto à classificação da MF obtido na segunda etapa foi maior do que aquele observado na primeira etapa, embora sem significância.

No que se refere à comparação entre ambos os julgamentos de uma mesma avaliadora, verificou-se aumento do índice kappa para a avaliadora 3, porém houve uma diminuição deste índice para as avaliadoras 1 e 2. Estas diferenças não foram significantes.

### Correlação entre o tamanho do orifício velofaríngeo e a mímica facial

A análise do coeficiente de correlação de Spearman revelou correlação positiva significativa moderada entre a mímica facial e a medida da área do orifício velofaríngeo tanto na primeira etapa da análise (p<0,01; r=0,550) quanto na segunda (p<0,01; r=0,553).

## DISCUSSÃO

Na prática clínica, bem como em pesquisas bem delineadas, cada vez mais, inúmeros ensaios têm sido testados na tentativa de se desenvolver métodos que resultem em maior concordância e confiabilidade de resultados, principalmente em procedimentos que envolvam avaliações perceptivas de natureza subjetiva. Atenção especial se dá aos métodos de classificação e escalas de avaliação de sintomas de fala tais como ressonância, inteligibilidade, qualidade vocal e articulação^(2,10-14)^.

Ainda que de grande importância na avaliação da fala de indivíduos com DVF, o mesmo destaque não é extensivo à mímica facial. Embora seja um importante indicador da DVF, nenhuma proposta para classificação da mímica facial foi, até então, publicada na literatura. Sua análise baseia-se na visualização direta do falante e usualmente, é classificada por escalas numéricas ordinais ou por escalas binárias, constituídas por presença ou ausência^(15)^.

A proposta de classificação da mímica facial utilizada no presente estudo baseou-se na experiência clínica de seus autores. Fez-se, então, uma breve explanação sobre os critérios utilizados para a análise das amostras na segunda etapa do estudo, a fim de verificar sua influência sobre os resultados.

Ao analisarmos os resultados da concordância intra-avaliadores, após a apresentação desta nova proposta, verificou-se redução do índice de concordância. Diversos fatores internos e externos, podem interferir em julgamentos subjetivos tal qual o do presente estudo, motivo pelo qual a avaliação perceptiva está sujeita à variabilidade mesmo quando é realizada por avaliadores experientes. O estado físico e emocional ao avaliar as amostras, bem como o padrão interno de cada avaliador, que é, também, instável podem ter contribuído para a variabilidade das respostas, mesmo em se tratando de avaliadores experientes na avaliação e tratamento de pacientes com FLP. Sabe-se que o padrão interno de um avaliador é desenvolvido ao longo de sua experiência com os diversos sintomas de fala em seus diferentes graus e, essas informações ficam armazenadas em sua memória^(10)^. Especula-se que, ao se confrontarem com uma proposta diferente da usualmente utilizada e, mais criteriosa no que se refere a localização do movimento compensatório, os padrões internos adquiridos ao longo de sua experiência (conceito de sintoma leve, moderado e grave) para este tipo de avaliação, acabaram por se agregar à nova maneira de avaliar, dificultando a repetição das respostas.

O mesmo, entretanto, não ocorreu com a análise interavaliadores. Nessa análise, verificou-se que o índice de concordância aumentou consideravelmente, após a apresentação da proposta de classificação, o que fala a favor da nova abordagem, principalmente no uso em estudos clínicos e em avaliação de resultados inter centros.

Esta maior concordância após o treinamento pode ser explicada pelo fato de que, nesta análise, os avaliadores utilizaram as descrições de referências pré-estabelecidas, ou seja, contaram com um modelo a ser seguido durante as avaliações. Isto, somado ao fato de que as avaliadoras realizaram a classificação logo após o treinamento, atesta a utilização destes procedimentos em análises subjetivas.

O fato de não ter sido realizado um treinamento das avaliadoras e não terem sido pré-estabelecidos critérios de análise da mímica facial na primeira etapa do estudo, como foi feito na segunda etapa, pode ser considerada uma limitação do estudo. Tal metodologia foi adotada a fim de evitar uma possível influência de um treinamento prévio, sobre os resultados da avaliação do sintoma feita seguindo os padrões internos e critérios utilizados na rotina clínica das avaliadoras.

A correlação do julgamento da mímica facial com a dimensão do orifício velofaríngeo aferido pela avaliação instrumental objetiva, em ambas as etapas, mostrou-se positiva e significante indicando que, quanto maior a intensidade de movimento durante a mímica facial, maior o tamanho da falha velofaríngea.

O emprego da rinomanometria para a avaliação da DVF, por se tratar de um método objetivo que determina o tamanho real da falha velofaríngea (*gap*, em inglês) é de grande importância na prática clínica. Por essa razão, um grupo de pesquisadores no Brasil tem utilizado este instrumento para elaborar propostas de protocolos de avaliações das características de fala de pacientes com DVF, cujos resultados, são baseados em informações, também, advindas deste instrumento^(2,3)^.

Espera-se, com os resultados destes estudos, que profissionais que não têm acesso a este tipo de equipamento possam, a partir das características perceptivas de fala, prever o tamanho da falha velofaríngea, possibilitando assim, uma conduta mais segura e efetiva.

## CONCLUSÃO

A proposta de julgamento visual da mímica facial baseada em critérios padronizados mostrou-se efetiva na determinação do sintoma e confiável no diagnóstico da gravidade da DVF. A correlação significativa entre os métodos perceptivo e instrumental de avaliação sugere que a mímica facial pode ser utilizada na previsão do fechamento velofaríngeo.
